# Determining the reliability of Artificial Intelligence programs in medical faculty board exams

**DOI:** 10.12669/pjms.41.12.12855

**Published:** 2025-12

**Authors:** Arif Keskin, Tayfun Aygun

**Affiliations:** 1 Arif Keskin, Department of Anatomy, Faculty of Medicine, Giresun University, 28100, Giresun, Turkiye; 2 Tayfun Aygun, Department of Anatomy, Faculty of Medicine, Giresun University, 28100, Giresun, Turkiye

**Keywords:** Anatomy, Artificial Intelligence, ChatGPT, Gemini, Copilot

## Abstract

**Objective:**

In recent years, artificial intelligence (AI) applications have become widespread in many fields, including medical education. This study aimed to examine the reliability of widely used generative AI programs, such as Microsoft Copilot, Google Gemini and OpenAI ChatGPT, by evaluating the accuracy of first- and second-year medical students’ responses to anatomy questions on mid-term board, final and make-up exams.

**Methodology:**

Total 286 anatomy questions from the 2023-2024 academic year by first and second semester medical school students in anatomy, 222 with analysis reports were included in the study. The difficulty levels of the questions were divided into four groups (very difficult, difficult, medium, easy) based on students’ correct answer rates. The same questions were then posed to three AI applications. The data were analyzed by SPSS-version 27.

**Results:**

According to the findings, Copilot, ChatGPT and Gemini achieved significantly higher accuracy compared to students, with 97.7% accuracy, 94.4% accuracy and 86.5% accuracy, respectively. However, Gemini and ChatGPT remained similar to students, particularly on very challenging questions. Gemini did not perform as well on questions requiring basic knowledge (first year) as on questions requiring clinical interpretation (second year).

**Conclusion:**

While the study found that AI applications provide higher accuracy compared to students, systems that fail to achieve 100% accuracy are not suitable for unrestricted and unsupervised use in critical basic medical sciences like anatomy. Because AI models lack clinical reasoning and human experience, they should be used only as supplementary educational tools and integrated in a controlled manner to enhance student success.

## INTRODUCTION

Anatomy provides fundamental knowledge to various fields of medicine. Therefore, it is an indispensable discipline in undergraduate medical education.^1^ Due to its complex and vital concepts, various educational methods are used in teaching anatomy. These methods help reinforce anatomical knowledge, which is often memorized and easily forgotten. Research highlights that the integration of advanced artificial intelligence (AI) into anatomy education represents a transformative trend that significantly enhances students’ learning experiences.^2^,^3^

Today, generative AI programs are driving significant changes in various fields, from healthcare to education.^4^ AI programs stand out with their numerous capabilities and functionalities. AI’s ability to present desired information from complex datasets in a human-like manner attracts students’ attention.^5^,^6^ Academic interest and expectations regarding the potential benefits of AI applications in medical education are growing.^7^,^8^ Testing the evidence-based effectiveness and applicability of AI applications is crucial for their systematic integration into future medical education curricula. This research aimed to assess the effectiveness of AI use in medical education using objective parameters by analyzing the accuracy rates of AI-based chatbot systems’ responses to anatomy questions in comparison with student performance.

This is a pioneering study evaluating exam performance in the context of medical education. By demonstrating the utility of AI as a complementary tool for anatomy, a fundamental medical science course requiring memorization, it sheds light on the future integration of AI into educational materials and exam preparation. Furthermore, revealing the performance differences between AI grades and questions of varying difficulty levels provides guidance on how these technologies can be adapted to medical education. In this context, the study makes an interdisciplinary contribution to both education and information technology.

## METHODOLOGY

This study examined the questions taken by first and second semester medical school students in anatomy, final and make-up exams during the 2023-2024 academic year.

### Ethical Approval:

Before starting the study, Karadeniz Technical University Health Sciences Scientific Research Ethics Committee approval (Ref. No.: E-13562490-050.01.04-633635; dated March 17, 2025) was obtained.

Anatomy questions from all exams taken by students were recorded. Exam evaluation reports prepared by the medical school assessment and evaluation board were accessed after each exam. These reports include the number of options provided by students for each question and the exam’s question analyses. Using the data from the evaluation reports, questions were classified according to difficulty level based on the percentage of correct answers.

### Classification

- Questions with a correct answer rate between 0 and 24.99% were classified as very difficult.
- Questions with a correct answer rate between 25 and 49.99% were classified as difficult.
- Questions with a correct answer rate between 50 and 74.99% were classified as moderate.
- Questions with a correct answer rate between 75 and 100% were classified as easy.

### Inclusion criteria:

- Questions prepared by the anatomy department, included in all exams, were recorded.
- Free access to ChatGPT-4 (OpenAI; San Francisco, CA), Gemini (Google, Mountain View, California, United States) and Copilot (Microsoft, Redmond, WA) chatbots was provided on a computer.

### Exclusion criteria:

- Questions containing visuals such as figures and pictures.
- Questions that were discarded in the evaluation reports were excluded from the study.

Before the questions were asked, a separate prompt was entered in the text section of the programs: “I am a faculty member in the anatomy department of the medical school. I will be asking you multiple-choice questions in the field of anatomy. I want you to tell me which answer is correct.” The programs’ responses to the questions were recorded. After comparing them with the exam answer keys, the answers were grouped. Due to the programs’ potential memory retention, a new chat session was opened for each question and the same prompt was entered.

### Statistical analyses:

Data were analyzed using IBM SPSS V27 (Statistical Package for Social Sciences, NY, USA) package program. Descriptive statistics of the evaluation results were given as number and percentage for categorical variables, mean and standard deviation for numerical variables. The compliance of the groups with normal distribution was determined by Kolmogorov-Smirnov test. Comparisons of numerical variables between two independent groups were evaluated with Student-t Test when normal distribution condition was provided and Mann-Whitney U test when it was not provided. Comparisons of numerical variables between more than two independent groups were determined with Anova test. Chi-square test was used to compare categorical variables of independent groups. Statistical alpha significance level was accepted as p<0.05.

## RESULTS

During the 2023-2024 academic year, first-year medical students participated in six exams, while second-year students took seven exams. A total of 286 anatomy questions were administered in these exams. Since exam analysis reports were not available for the first- and second-year Committee I exams, 64 questions were excluded from the evaluation. Among the remaining 222 questions with available analysis reports, there was no significant difference in the number of questions across difficulty levels (p=0.48).

The classification of questions by difficulty level was based on the number of correct responses given by students. The proportional differences in correct response rates among the four difficulty groups were statistically significant (p<0.001). The classification of questions by difficulty level and the corresponding student accuracy rates are presented in Table-I.

**Table-I:** Number of Correct Answers, Mean, Maximum and Minimum Scores by Difficulty Level.

		n	Mean	Std. Dev.	Median	Min.	Max.	p
Term I	Very Difficult	7	18.85	2.48	17.39	15.86	21.92	<0,001
	Difficult	23	39.39	7.56	41.78	26.09	48.97	
	Medium	29	64.16	8.41	65.75	50.68	73.91	
	Easy	30	85.28	7.33	85.91	75.17	100.00	
	Total	89	61.31	22.86	65.75	15.86	100.00	
Term II	Very Difficult	8	20.92	5.17	22.65	8.89	24.49	<0,001
	Difficult	31	40.48	8.97	41.10	26.67	73.29	
	Medium	57	63.32	7.60	63.70	50.68	84.93	
	Easy	37	84.67	6.54	83.92	75.56	96.53	
	Total	133	61.38	20.28	63.70	8.89	96.53	

Among the first year questions classified by difficulty level, CoPilot outperformed the other AI programs. It was able to generate correct answers not only for moderate and easy questions but also for very difficult and difficult ones. Gemini struggled with very difficult questions; however, its performance improved as the questions became easier. Nevertheless, this difference was not statistically significant (p=0.285). ChatGPT, similar to students, faced challenges with very difficult questions. However, as the difficulty level decreased from very difficult to easy, the number of correct responses increased significantly (p<0.05). The number of correct and incorrect responses provided by AI programs based on question difficulty is presented in Table-II.

**Table-II:** Performance of AI Programs in Answering First Year Questions.

First Year		Very Difficult n (%)	Difficult n (%)	Medium n (%)	Easy n (%)	P
CoPilot	Incorrect	0 (0)	1 (4.3)	1 (3.4)	0 (0)	0.682
	Correct	7 (100.00)	22 (95.7)	28 (96.6)	30 (100.00)	
Gemini	Incorrect	3 (42.9)	8 (34.8)	6 (20.7)	5 (16.7)	0.285
	Correct	4 (57.1)	15 (65.2)	23 (79.3)	25 (83.3)	
ChatGPT	Incorrect	3 (42.9)	1 (4.3)	2 (6.9)	0 (0)	0.001
	Correct	4 (57.1)	22 (95.7)	27 (93.1)	30 (100.00)	

CoPilot, Gemini and ChatGPT demonstrated similar response accuracy rates for the second year questions. They struggled with very difficult questions, achieving a success rate of 75%. As the questions became easier, their performance improved significantly and this difference was statistically significant (p<0.05). The performance rates of CoPilot, Gemini and ChatGPT in answering second year questions based on difficulty level are presented in Table-III.

**Table-III:** Number of Correct and Incorrect Responses by AI Programs for Second Year Questions Based on Difficulty Level.

Second Year		Very Difficult n (%)	Difficult n (%)	Medium n (%)	Easy n (%)	P
CoPilot	Incorrect	2 (25)	2 (6.5)	0 (0)	1 (2.7)	0.005
	Correct	6 (75)	29 (93.5)	57 (100.00)	36 (97.3)	
Gemini	Incorrect	2 (25)	1 (3.2)	0 (0)	0 (0)	0.000
	Correct	6 (75)	30 (96.8)	57 (100)	37 (100.00)	
ChatGPT	Incorrect	2 (25)	2 (6.5)	1 (1.8)	1 (2.7)	0.025
	Correct	6 (75)	29 (93.5)	56 (98.2)	36 (97.3)	

Gemini demonstrated a significantly higher performance in correctly answering second year questions compared to first year questions (p<0.05). In contrast, CoPilot and ChatGPT had similar accuracy rates across first-second year. The accuracy rates of AI programs in answering questions based on student class are presented in Table-IV.

**Table-IV:** Accuracy Rates of Artificial Intelligence Programs According to Student Classes.

Artificial Intelligence	First Year	Second Year	p
CoPilot	87 (97.75)	128 (96.24)	0.527
Gemini	67 (75.28)	130 (97.74)	0.000
ChatGPT	83 (93.26)	127 (95.49)	0.471

The performance of AI programs in answering first year questions showed a statistically significant difference (p<0.05). Gemini was notably the least successful, while CoPilot and ChatGPT demonstrated similar accuracy rates. Although the success rates of AI programs in answering second year questions varied, these differences were not statistically significant (p>0.05).

## DISCUSSION

The findings of this study indicate that AI-based chatbot systems (Copilot 97.7%, ChatGPT 94.4%, Gemini 86.5%) exhibit significantly higher accuracy rates than students (61.35%) on anatomy questions. However, the fact that Gemini and ChatGPT performed similarly to students on very difficult questions reveals that these systems have limitations in situations requiring complex anatomical knowledge. The fact that ChatGPT outperforms older versions demonstrates its continued development and that future versions may deliver even better results. As Khan et al.^9^ emphasized, the evolution of ChatGPT could reshape medical education and clinical management.

Studies evaluating ChatGPT’s performance on medical exams in the literature report similar performance degradation on difficult questions.^7^,^10^ Our findings support this observation, showing that ChatGPT dropped to 57% accuracy on very difficult first-grade questions. While Mavrych et al.^11^ reported ChatGPT to be the most successful model in their study covering gross anatomy questions, Copilot performed the best in our study. This difference can be explained by the fact that the questions were asked in Turkish and Copilot’s superior language processing capacity.^12^

A notable finding is that Gemini underperformed first-grade questions (75.28%) but showed a dramatic improvement in second-grade questions (97.74%). This may be attributed to Gemini’s multimodal training dataset and superiority in questions requiring clinical interpretation.^13^ Aksoy and Arslan,^14^ also reported that Gemini underperformed Copilot in a comparison of emergency medicine questions, which is consistent with our findings.

The primary hypothesis of this study was that AI systems can be used as a reliable resource in anatomy education. Our findings suggest that this hypothesis is partially correct; although AI systems exhibit high accuracy rates, they cannot achieve 100% accuracy, making them unsuitable for unsupervised use.

### Strengths:

This study is one of the first to comparatively examine the accuracy rates of AI programs using real exam questions administered to medical students. Classifying questions by difficulty allowed for a detailed assessment of the knowledge level and analytical capabilities of AI applications. Furthermore, starting a new session for each question to prevent the models from being influenced by past data increases the methodological reliability of the study. Using real student data also provides a high level of validity for the study.

### Limitations:

A key limitation of the study is that our findings may vary over time due to the constant updating of AI systems. Asking the same questions on different dates may yield different accuracy rates. Furthermore, only evaluating questions in Turkish poses another limitation.

## CONCLUSION

This study systematically evaluated the performance of three different AI-based language models (ChatGPT, Gemini and Copilot) on medical school anatomy exams. The findings indicate that all chatbots generally achieved high student accuracy rates but experienced limitations with complex questions.

The study findings suggest that AI models can be used as a complementary tool in anatomy education, particularly in areas requiring memorization and knowledge integrity. However, it should be noted that unrestricted and unsupervised use of these systems, especially in critically important basic medical sciences, can lead to inaccurate learning. Future research should focus on developing standardized protocols for integrating AI systems into educational curricula and prospectively evaluating their effects on long-term educational outcomes. Therefore, AI applications in current medical education should be considered for limited use only as complementary resources.

### Authors’ Contribution:

**AK and TA:** Conceived, designed, did statistical analysis & editing of the manuscript and are responsible for the integrity of the research.

**AK and TA:** Did data collection, critical review and manuscript writing.

**AK and TA:** Literature search, Did review and gave final approval of the manuscript.
